# Case–control association analysis of Dopamine receptor polymorphisms in alcohol dependence: a pilot study in Indian males

**DOI:** 10.1186/1756-0500-6-418

**Published:** 2013-10-17

**Authors:** Pushplata Prasad, Atul Ambekar, Meera Vaswani

**Affiliations:** 1 National Drug Dependence Treatment Centre, All India Institute of Medical Sciences, New Delhi 110029, India; 2 Present Address: TERI-Deakin Nano-biotechnology Centre, Biotechnology and Management of Bio-resources Division, The Energy and Resources Institute, India Habitat Centre, Lodhi Road, New Delhi 110003, India

**Keywords:** Alcohol dependence, Dopamine receptors, Polymorphisms, Case–control study, Genetic association

## Abstract

**Background:**

Brain imaging studies and knock-out animal models have derived substantial abetment for dopamine receptor (DR) subtypes as potential candidates in susceptibility to addictive disorders, including alcohol dependence (AD). Various association studies that compared the frequencies of alleles of the dopamine D1, D2, D3 and D4 receptor genes between alcohol dependent and control subjects have produced suggestive results, though some of them are discordant in nature. In the absence of genetic data from Indian population, we evaluated genetic association of three polymorphisms namely rs4532 in *DRD1*, rs6280 in *DRD3* and 120 bp duplication in 1.2 kb upstream region of *DRD4* with AD.

**Methods:**

A total of 90 cases (alcohol dependent males) and 122 age and ethnicity matched healthy male controls were recruited in the study by following DSM-IV criteria. Three polymorphisms, namely rs4532 in *DRD1*, rs6280 in *DRD3* and 120 bp duplication in 1.2 kb upstream region of *DRD4* were selected (based on minor allele frequency and available literature) for genotyping by PCR-RFLP/LP method. Allele and genotype frequencies of these genetic markers were compared using Pearson’s *χ*^2^ test followed by risk assessment using odds ratio. Statistical analysis of clinical parameters such as AUDIT scores of case subjects was also performed.

**Results:**

Statistically significant associations of polymorphisms in *DRD1* and *DRD4* with alcoholism were found.

**Conclusions:**

Our results underscore that genetic variations in dopamine receptors D1 and D4 may influence genetic predisposition to alcoholism. Unavailability of comparative data from Indian population and small sample size necessitate replication of results in an independent cohort.

## Background

Alcohol dependence is a chronically relapsing addictive disorder which is characterized by compulsive and continued intake of substance of abuse despite negative consequences [1]. A key factor in development of addiction is the reinforcing capacity of drug of abuse that brings about complex changes in brain leading to neuronal adaptations [2]. Alcohol influences reward pathway through mesolimbic dopamine system in the brain. Empirical evidences from animal studies suggest that alcohol enhances the firing rates of dopaminergic neurons in the ventral tegmental area (VTA). This is followed by increased release of dopamine in the nucleus accumbens (NAc) which forms the basis of compulsive-reinforcing properties of alcohol and the reward mechanism [3-7]. Varying densities of both the dopamine receptors and transporters in humans have been found to be associated with AD [8,9].

Multiple small genes and their interaction with environment cumulatively influence development of alcohol dependence [10-12]. Family, adoption and twin studies suggest that approximately 50-60% risk to AD could be attributed to genetic components [13]. Of the various pathways and genes, genetic polymorphisms in dopamine receptor (DR) subtypes are believed to influence the development and/or severity of alcoholism. Genetic association of dopamine D2 receptor (DRD2) gene with alcohol dependence is most widely studied [14,15]. We have previously reported significant association of -141C Ins/Del polymorphism in *DRD2* with AD in a north-Indian cohort [16]*.* In the present study we have investigated the association of *DRD1, DRD3 & DRD4* gene polymorphisms with AD in a population of north-Indian origin.

Dopamine receptor −1 *(DRD1)* is considered a candidate gene in alcohol dependence, particularly with regard to its role in the prefrontal cortex in modulation of cognitive processes [17]. A recent study reported that miR‒382 which directly targets *DRD1* in the NAc plays a crucial role in mediating the behavioral responses to alcohol [18]. Among the different polymorphisms of the DRD1 gene, none have been associated with alcohol dependence. However, rs4532 SNP, a -48A⁄G Dde I polymorphism in the 5′-UTR of the gene, displayed a modest role in a large set of phenotypes including addictive behaviors [19].

Dopamine receptor D3 (*DRD3*) is highly expressed in the limbic area [20] in the NAc and influences the reward process of addiction behavior. *DRD3* is not involved in the direct reinforcing effects of drugs of abuse, but seems to contribute to the motivational aspects and/or the effects of cues over behaviors [21]. In addition, depleted D2/D3 receptors levels in human striatum found by brain imaging studies have become an evident marker of addiction in patients, even during periods of withdrawal [22]. However, genetic association studies carried out in different populations report conflicting results on influence of genetic polymorphisms of *DRD3* on AD [23-27].

Only a few genetic association studies have been conducted with *DRD4* and *DRD5*. These receptors are expressed in cortex and hippocampus and may modulate some response to drugs. Recent findings suggest that *DRD4* may be involved in cue reactivity [28].

Incidence of alcohol dependence (AD) is increasing with the changing lifestyle. A lifetime prevalence of 6.5% in India has been estimated [29]. Thus, studying the bio-genetic factors for AD in Indian population becomes imperative.

## Methods

### Study population

Details of the study population have been published elsewhere [16]. Briefly, ethical committee clearance from the All India Institute of Medical Sciences (AIIMS, New Delhi) and written informed consent from the study participants were obtained prior to sample collection. Using the DSM-IV criteria [30], clinical assessments were carried out by a qualified Psychiatrist. One hundred and forty male alcohol dependent subjects attending the outpatient department (OPD) at National Drug Dependence Treatment Centre, AIIMS, were screened. Out of these, 37 were polysubstance users and 13 had co-morbid depression/anxiety/or schizophrenia, and therefore, were excluded from the study. Remaining 90 unrelated outpatients with alcohol dependence, in the age range of 18–60 years, were enrolled as cases. A total of 122 unrelated healthy male employees of the hospital, without any history of substance use (except nicotine) were included as controls in the study. Since nicotine use is widely prevalent among males in India, neither case nor controls were excluded on the basis of their nicotine use. Subjects with a current diagnosis of dependence or abuse of other substances except nicotine, a current psychiatric diagnosis, and evidence of severe neurologic or psychiatric disorders, mental retardation were excluded from the study.

All patients were assessed for alcohol use parameters using the Alcohol Use Disorders Identification Test (AUDIT) [31] and a semi-structured questionnaire. The semi-structured questionnaire included items on clinical details like ethnicity, family history, age at first use of alcohol, quantity of alcohol consumption (g/day), duration of alcohol use, duration of alcohol dependence, age at onset of dependence, presence/absence of delirium and any other psychiatric or physical illness. The same semi-structured questionnaire was used for assessment of the control population as well. The laboratory assessments included liver function tests (LFT) such as serum proteins, albumin, bilirubin, glutamic oxaloacetic transaminase (SGOT), glutamic pyruvic transaminase (SGPT), and gamma-glutamyltransferase (GGT), were estimated on autoanalyser using biochemical kits from Boehringer Mannheim kits (Germany). The study was conducted in accordance with worldwide good clinical practice (GCP) standards and confirmed to acceptable ethical standards as outlined by local requirements and the Declaration of Helsinki (World Medical Association, 1989).

### Genetic analysis

Phenol chloroform organic extraction method [32] was employed for DNA isolation from peripheral blood lymphocytes. Three polymorphisms namely rs4532 in *DRD1*, rs6280 in *DRD3* and 120 bp duplication in 1.2 kb upstream region of *DRD4* were selected based on prior published genetic association reports, information content, minor allele frequency (MAF) and validation evidence. SNP genotyping was done following polymerase chain reaction (PCR)-restriction fragment length polymorphisms (RFLP)/length polymorphism (LP) approach reported previously [33].

### Statistical analysis

Clinical variables were compared between alcohol dependent and control subjects using *χ*^2^ test for nominal variables and student’s *t*-test/Mann Whitney’s *U* test for continuous variables.

Hardy-Weinberg equilibrium (HWE) was calculated for the genetic polymorphisms by *χ*^2^ test. Allelic and genotypic associations of SNPs were evaluated by Pearson’s *χ*^2^ test followed by risk assessment using odds ratio and 95% confidence of interval (CI) computation. Power of the sample size for each of the SNPs was calculated using PAWE software version 1.2 [34,35].

## Results

### Clinical analysis

Demographic and Clinical characteristics of the study population are reported previously [29]. On almost all demographic parameters, the control group was largely similar to the case group. However, larger proportion of AD subjects had education only up to primary school (i.e. five years of formal schooling). The values for various clinical parameters such as audit score, alcohol intake (g/day) SGOT, SGPT, GGT, cholesterol, and triglycerides (TG)] were significantly higher (P < 0.01) among alcohol dependent cases as compared to the control group [Table 1].

**Table 1:** Demographic and clinical parameters significantly different between the case and control groups (presented as Mean; SD/SE)

**Characteristics**	**Case**	**Controls**	**OR (95% CI)**
Audit Score	32.12 ± 5.59	1.043 ± 1.58	31.07 (29.85-32.29)
Alcohol intake (g/day)	183.89 ± 104.54	2 ± 0.5	79.35 (78.91-79.78)
Total bilirubin (mg/dl)	1.96 ± 0.78	0.7 ± 0.2	1.26 (1.05-1.46)
SGOT (U/l)	84.21 ± 27.76	27.02 ± 09.71	57.19 (50.95-63.42)
SGPT (U/l)	83.13 ± 26.29	28.30 ± 11.49	55.11 (48.95-61.27)
GGT (U/l)	210.02 ± 42.02	24.95 ± 11.88	185.07 (175.88-194.26)
TG (mg/dl)	198.58 ± 108.01	121.59 ± 11.8	76.99 (54.47-99.50)

### Genetic analysis

Genotypic and allelic frequencies of two SNPs in DRD1 and DRD3 were in agreement with the Hardy-Weinberg equilibrium (HWE). However, ins/del polymorphism in DRD4 deviated from HWE. Allele and genotype frequencies of SNPs in *DRD1, DRD3,* and *DRD4* and their association status with AD along with power of sample set are presented in Table 2. A significant allelic and genotypic associations of −48 A > G SNP of *DRD1* and −120 Ins/Del polymorphism of *DRD4* were observed with alcohol dependence. No association between Ser9Gly SNP of *DRD3* and alcoholism was observed.

**Table 2:** **Allele and genotype frequencies of polymorphisms in *DRD1, 3, 4 *and their association status with alcohol dependence**

**SNPs**	**Genotypes**			***χ***^**2 **^**(P)**	**Alleles**		***χ***^**2 **^**(P)**	**OR (95% CI)**	**Power (G%)**
**D1 -48A > G**	**AA**	**AG**	**GG**		**A**	**G**			
**Cases**	0.14	0.49	0.37	**7.65 (0.02)**	0.38	0.62	**3.96 (0.04)**	**1.77 (1.01-3.10)**	76%
**Controls**	0.29	0.46	0.25		0.52	0.48			
**D3 Ser9Gly**	**CC**	**CT**	**TT**		**C**	**T**			
**Cases**	0.29	0.62	0.09	5.48 (0.07)	0.60	0.40	0.51 (0.47)	1.22 (0.7-2.15)	5%
**Controls**	0.30	0.50	0.20		0.55	0.45			
**D4 120 Ins/Del**	**Ins/Ins**	**Ins/Del**	**Del/Del**		**Ins**	**Del**			
**Cases**	0.45	0.49	0.06	**11.4 (0.00)**	0.72	0.28	**6.14 (0.01)**	**2.10 (1.17- 3.79)**	86%
**Controls**	0.39	0.31	0.30		0.55	0.45			

## Discussion

Alcohol dependence is a heterogeneous disorder associated with a spectrum of psychiatric and medical problems. A complex interaction of genetic and environmental factors underlies the biogenesis of AD. Dopamine being an important neurotransmitter in the reward pathway, the associated genes and their polymorphisms are of significant interest. Genes encoding dopamine receptor (DR) subtypes have received considerable attention as a potential candidate that may affect susceptibility to alcoholism [36]. There is strong support for the dopamine hypothesis of the reinforcing effects of ethanol and development of alcohol dependence [2]. We tested genetic association of three polymorphisms in the dopaminergic pathway genes namely rs4532 in *DRD1*, rs6280 in *DRD3* and a 120 bp duplication in 1.2 kb upstream region of *DRD4* with alcohol dependence among male subjects from the North India.

rs4532 present in 5′ UTR of *DRD1* is a widely investigated polymorphism for genetic association with mental health and addiction. This SNP has shown significant association with alcohol dependence and associated disorders like bipolar disorder [37,38] and novelty seeking, persistence and harm avoidance [39,40]. In line with such genetic associations, rs4532 showed significant association with alcohol dependence in the present study. Significant power of association further indicates that -48A > G SNP could be a predisposing factor in North Indian subjects. Further, this regulatory SNP has been reported to be significantly associated with severity of alcohol-related problems, as measured by the AUDIT score in a gene dose-dependent manner [40], high MAST score [19], and elevated sensation seeking scores [39]. In concurrence to above, significantly higher audit score, higher alcohol consumption, followed by impairment of liver function tests were seen in case subjects with G allele or GG genotype. Such observations may support the hypothesis of Batel et al. [9] that *DRD1* could play an indirect role in alcohol dependence through severity rather than presence or absence of alcohol dependence.

*DRD3* gene is located in the limbic area, in particular in the nucleus accumbens, which plays a significant role in the reward process of addiction behavior [27]. The BalI polymorphism (rs6280; Ser9Gly) in *DRD3* is a point mutation that results in substitution of a glycine for serine residue in the extracellular receptor N-terminal domain [41]. Genetic analyses across different populations report inconsistent association of this non-synonymous SNP with alcoholism. Positive associations [27,42] supported involvement of *DRD3* in the development of addiction to alcohol. However, large number of studies in French, Korean and Caucasian populations reported no association of rs6280 [23,36,43] with AD, suggesting that the SNP may not be important in the genesis of alcoholism. The present study did not record any association of rs6280 with AD. This is in complete agreement with negative findings in different populations as indicated above.

The *DRD4* polymorphism 120 bp tandem duplication, located 1.2 kb upstream of the initiation codon has been well studied in context of attention deficit hyperactivity disorder (ADHD). This repeat sequence has been hypothesized to have a potential role in transcription regulation. McCracken et al. (2000) reported significant association with ADHD and indicated that duplication could be a risk factor by decreasing the expression of the DRD4 gene [44]. Further, transcription regulation was suggested to be mediated by the presence of consensus-binding sequences for several transcription factors in this region [45]. However, the genetic association studies involving the *DRD4* polymorphism and ADHD have been dissonant [46-48]. Association of *DRD4* with alcohol dependence has not been largely investigated. A study by Rogers et al. (2004) reported an association with novelty seeking in bipolar and alcoholic families [49]. We therefore, genotyped this polymorphism in Indian population and found a significant association with -120Ins (duplication) allele conferring approximately two times higher risk to alcohol dependence as compared to the control population. Susceptibility conferred by the duplication allele may draw support from the genetic association study of this polymorphism with ADHD where the duplication allele is reported to confer risk by negatively regulating *DRD4* transcription [44]. However, the −120 Ins/Del polymorphism in *DRD4* deviated from HWE in this study and therefore, the association result obtained for this marker must be interpreted with caution.

## Conclusions

Exploration of the underlying genetic factors in alcohol dependence in humans as well as in animal models has made great strides over the past four decades, and newer and finer approaches are being continuously evaluated. The present *post-hoc* genetic analysis of three dopamine receptor genes in AD indicates that the polymorphisms in the *DRD1* and *DRD4* may influence susceptibility to AD in the subjects of north Indian origin.

## Competing interests

The authors declare that they have no competing interests.

## Authors’ contributions

All authors have read and approved the final manuscript. PP was involved in conceptualization of the project, study design, carried out molecular genetics and statistical analyses, compiled the data, wrote the manuscript. AA was the clinical investigator involved in study design, defining exclusion and inclusion criteria of study subjects and was mainly responsible for identification of study subjects; MV was the principal scientist and coordinator of the project, involved in study design, critical inputs and finalization of the manuscript.
